# Sensorimotor Gating Depends on Polymorphisms of the Serotonin-2A Receptor and Catechol-*O*-Methyltransferase, but Not on Neuregulin-1 Arg38Gln Genotype: A Replication Study

**DOI:** 10.1016/j.biopsych.2009.05.007

**Published:** 2009-09-15

**Authors:** Boris B. Quednow, Anne Schmechtig, Ulrich Ettinger, Nadine Petrovsky, David A. Collier, Franz X. Vollenweider, Michael Wagner, Veena Kumari

**Affiliations:** a Department of Psychiatry, University of Bonn, Germany; b University Hospital of Psychiatry, Division of Neuropsychopharmacology and Brain Imaging, University of Zurich, Switzerland; c Department of Psychology, Institute of Psychiatry and National Institute for Health Research (NIHR), Biomedical Research Centre for Mental Health, King's College London, London, United Kingdom; d Departments of Psychiatry and Psychology, Ludwig-Maximilians-University, Munich, Germany; e Social, Genetic and Developmental Psychiatry Research Centre, Institute of Psychiatry and NIHR Biomedical Research Centre for Mental Health, King's College London, United Kingdom

**Keywords:** 5-HT_2A_ receptor, acoustic startle response, polymorphism, prepulse inhibition, schizophrenia, sensorimotor gating, serotonin-2A receptor

## Abstract

**Background:**

Prepulse inhibition (PPI) of the acoustic startle response (ASR) is an operational measure of sensorimotor gating and a promising endophenotype of schizophrenia. We have recently shown that the linked serotonin-2A receptor (5-HT_2A_R) A-1438 G and T102C polymorphisms modulate PPI in schizophrenia patients. Moreover, it was shown that genetic variation in the catechol-*O*-methyltransferase (COMT) and the neuregulin-1 (NRG-1) proteins influences PPI in schizophrenia patients and healthy volunteers. Therefore, we aimed to replicate these results and investigated the impact of the related polymorphisms on PPI in healthy human volunteers.

**Methods:**

We analyzed the 5-HT_2A_R A-1438 G/T102C (rs6311/rs6313), the COMT Val158Met (rs4680), and the NRG-1 Arg38Gln (rs3924999) polymorphisms, assessing startle reactivity, habituation, and PPI of ASR in 107 healthy Caucasian volunteers.

**Results:**

Subjects homozygous for the 5-HT_2A_R T102C-T/A-1438 G-A allele showed increased PPI levels. In particular, male subjects with the COMT Met158Met-genotype also showed elevated PPI. The NRG-1 Arg38Gln genotype did not have a significant impact on PPI. Startle reactivity was not affected by any of the investigated polymorphisms.

**Conclusions:**

We confirmed in an independent sample of healthy volunteers that PPI is influenced by genetic variation in the 5-HT_2A_R gene. The influence of the COMT Val158Met genotype on PPI appears to be sex-specific. These results underscore the significance of the serotonin and dopamine systems in the modulation of sensorimotor gating.

Prepulse inhibition (PPI) of the acoustic startle response (ASR) is widely used as an operational measure of sensorimotor gating (1). PPI refers to the reduction of ASR magnitude when a distinctive nonstartling stimulus is presented 30–500 msec before a startling stimulus (2). It was proposed that the mechanism underlying PPI regulates sensory input by filtering out irrelevant or distracting stimuli to prevent sensory information overflow (1).

PPI was suggested as a promising endophenotype of schizophrenia for several reasons (3): 1) schizophrenia patients consistently show PPI deficits (4), 2) unaffected first-degree relatives of schizophrenia patients also exhibit decreased PPI levels (5,6), 3) the PPI deficit seems to be a trait marker of schizophrenia because it is already present in the prodromal phase of schizophrenia (7), 4) Inbred rodent studies and human twin studies suggested that PPI is heritable (8–10), and 5) PPI is measurable in a wide range of species in which PPI deficits could be artificially induced by environmental or pharmacologic manipulations. This offers the possibility to study the neurobiological basis of PPI in translational investigations (1).

Several single nucleotide polymorphisms (SNPs) have been reported to affect PPI strongly, thereby illuminating both the molecular mechanisms and genetic influences on sensorimotor gating. Initially, Hong *et al.* (11) found that the neuregulin-1 (NRG-1) Arg38Gln SNP modulates PPI in healthy volunteers and schizophrenia patients. Roussos *et al.* (12) followed with their finding that the catechol-*O*-methyltransferase (COMT) Val158Met SNP affects PPI in healthy male volunteers, a discovery which we recently replicated in a mixed-sex sample of schizophrenia patients (13). Additionally, we showed that the serotonin-2A receptor (5-HT_2A_R) A-1438 G and T102C SNPs (which are in complete linkage disequilibrium) are associated with PPI in schizophrenia patients (14). Most recently, it has been demonstrated that PPI also depends on the dopamine-D3 receptor Ser9Gly SNP (15). All of these SNPs have at some stage been suggested as susceptibility polymorphisms for schizophrenia (e.g., 16,17), but current meta-analyses do not support their role in etiology of the disease itself—with the exception of the 5-HT_2A_R A-1438 G polymorphism, which may have a small effect on the risk for schizophrenia (SchizophreniaGene: http://www.schizophreniaforum.org/res/sczgene) (18).

Replication is essential for establishing the credibility of genotype–phenotype associations (19). Therefore, we investigated the impact of four SNPs, which were previously linked to sensorimotor gating and schizophrenia, on PPI in a new and independent sample of healthy human volunteers: The linked 5-HT_2A_R T102C/A-1438 G, the COMT Val158Met, and the NRG-1 Arg38Gln polymorphisms.

## Methods and Materials

### Participants

One hundred seven healthy Caucasian volunteers (49.5% women; 24.3% smokers; mean age 26.2 ± 5.8 [SD] years, range: 18–43) were recruited through local advertisements in South London, United Kingdom. Participants were screened for the exclusion criteria of DSM-IV Axis I disorders using the Structured Clinical Interview for DSM-IV Disorders (SCID-I). Additional exclusion criteria were a history of head injuries, any known neurological abnormalities or systemic illness with known neurological complication, a first-degree relative with psychosis or obsessive-compulsive disorder, and a history of substance abuse or dependence. Ethical approval of the local ethics committee was obtained, and participants provided written informed consent.

### Genotyping

DNA was obtained from venal blood or buccal swabs using established procedures (20). 5-HT_2A_R A-1438 G (rs6311) and T102C (rs6313), COMT Val158Met (rs4680), and NRG-1 Arg38Gln (rs3924999) SNP genotyping assays were run as submicroliter polymerase chain reaction–based assays on Array Tape (http://www.douglasscientific.com) at PreventionGenetics (Marshfield, Wisconsin). They used an allele-specific polymerase chain reaction assay as described by Myakishev *et al.* (21) (rs6313, rs3924999) or InvaderPlus reactions from Third Wave Technologies (Madison, Wisconsin) (rs4680, rs6311). To improve genotyping reliability, many samples of similar DNA quality and concentration were genotyped at the same time. Genotyping was successful in 93.5% of subjects for 5-HT_2A_R A-1438 G and T102C, in 88.8% for COMT Val158Met, and in 95.3% for NRG-1 Arg38Gln SNP.

### Startle Response Measurement

Equipment, set up, PPI testing, and data acquisition and scoring procedures have previously been described in detail (22). Each examination began with a 4-min acclimation period of 70-dB background noise that was continued throughout the session. Participants received 49 white noise sound pulses at an intensity of 115 dB (duration of 40 msec) separated by variable intertrial intervals between 9 and 23 sec (mean = 15 sec). In 36 of the trials, the pulse was preceded by a 20-msec, 85-dB white-noise prepulse with stimulus-onset asynchronies (SOA) of 30, 60, and 120 msec (12 trials each). The initial trial was a pulse-alone (PA) trial, which was separated for further analysis. All following trials were presented in a pseudo-randomized order. The entire test session lasted approximately 16 min. To ensure that PPI was not influenced by smoking withdrawal, smoking ad libitum was permitted before testing (23). Trial exclusion and scoring criteria were identical to those used in previous studies (24). Subjects with response rejections > 50% were excluded from data analysis (*n* = 4).

### Statistical Analysis

Startle reactivity was assessed by the mean amplitude of the first block of PA trials and the mean amplitude of all PA trials. For the assessment of startle habituation, PA trials were divided each in four blocks. The calculation of the mean percent PPI and the habituation measures (percent habituation and linear gradient coefficient *b*) have been described in detail elsewhere (24).

All demographic data were analyzed by analysis of variance (ANOVA) with exception of frequency data. Frequency data were analyzed using χ^2^ tests. Given that sex (25) and smoking status (23) could affect PPI, these variables were introduced as covariates in all analyses of covariance (ANCOVA) of the psychophysiologic parameters independent of the statistical significance of the covariates. On the basis of significant main effects or interactions, Tukey honest significant difference (HSD) post hoc comparisons were performed. Given that we proposed directional hypotheses regarding the genotype effects on sensorimotor gating, statistical comparisons of the PPI data were carried out at a significance level set at *p* < .05 (two-tailed). Considering that we investigated four SNPs, all other confirmatory statistical comparisons were carried out at a Bonferroni-corrected significance level of *p* < .0125 (2-tailed). Within the Pearson Product-Moment correlation analyses, the significance level was set at *p* < .01 (two-tailed) to avoid accumulation of alpha error. Effect size calculations between two groups refer to Cohen's *d*. When post hoc tests of PPI data are reported, Cohen's *d* calculations based on pooled SOA conditions.

## Results

### 5-HT_2A_ T102C and A-1438 G Receptor Polymorphisms

As expected, the 5-HT_2A_R T102C and A-1438 G polymorphisms were in complete linkage disequilibrium (*r*^2^ = 1.0). Genotype frequencies were distributed in accordance with Hardy-Weinberg Equilibrium [HWE; χ^2^(1) = 1.1; *p* = .29]. The genotype groups did not differ regarding demographic variables, startle reactivity, and habituation measures (see Table 1). Moreover, startle latency and prepulse latency facilitation was not affected by 5-HT_2A_R genotype (data not shown).

**Figure 1:**
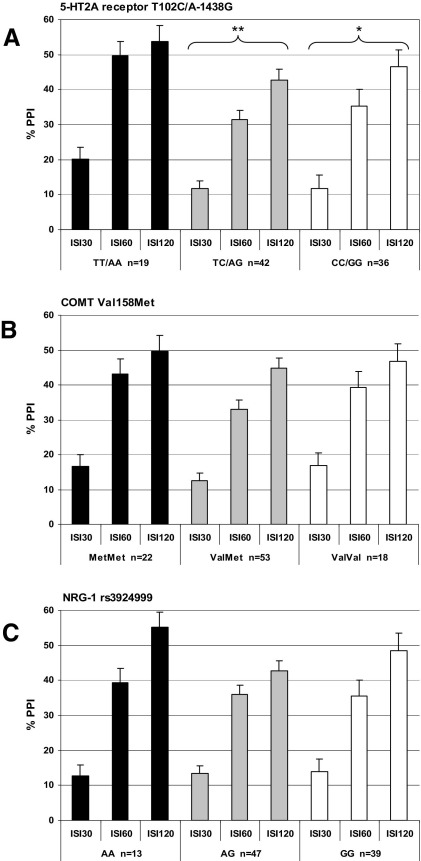
The effects of genotype on percent prepulse inhibition (PPI) of the acoustic startle response at prepulse (onset)-to-pulse (onset) intervals of 30, 60, and 120 msec in healthy human volunteers (means and SEM, adjusted for sex and smoking): **(A)** the completely linked serotonin-2A receptor (5-HT_2A_) A-1438 G and T102C receptor polymorphisms (Tukey honest significant difference post hoc test vs. TT/AA allele group: *p < .05, **p < .01), **(B)** the catechol *O*-methyltransferase (COMT) Val158Met polymorphism, and **(C)** the neuregulin-1 (NRG-1) Arg38Gln polymorphism.

A 3 × 3 (SOA condition × genotype) repeated-measures ANCOVA with sex and smoking as covariates revealed significant main effects for the factors SOA condition [*F*(2,91) = 7.7; *p* < .001; η^2^ = .14], sex [*F*(1,92) = 22.2; *p* < .001; η^2^ = .19], and genotype [*F*(2,92) = 5.0; *p* < .01; η^2^ = .10; see Figure 1A]. Tukey HSD post hoc tests revealed that homozygous carriers of the T102C-T/A-1438 G-A allele did show significantly higher PPI levels compared to homozygous T102C-C/A-1438 G-G (*p* < .05; *d* = .63) and the heterozygous T102C-TC/A-1438 G-AG variants (*p* < .01; *d* = .81). Homozygous T102C-C/A-1438 G-G carriers and heterozygous T102C-TC/A-1438 G-AG carriers did not differ in PPI. The main effect of SOA reflects the well-known nature of PPI to increase with rising SOA from 30 msec through 60–120 msec (26). The effect of sex points to the known fact that women have generally lower PPI levels than men [pooled SOA conditions: *F*(1,102) = 18.3; *p* < .001; η^2^ = .15] (25).

### COMT Val158Met Polymorphism

The COMT Val158Met genotype frequencies were distributed in accordance to the HWE [χ^2^(1) = 1.9; *p* = .17]. The three genotype groups did not differ in demographic characteristics, pooled PPI scores, startle reactivity, and habituation measures (Table 2). Startle latency measures were also not influenced by COMT genotype (data not shown).

**Figure 2:**
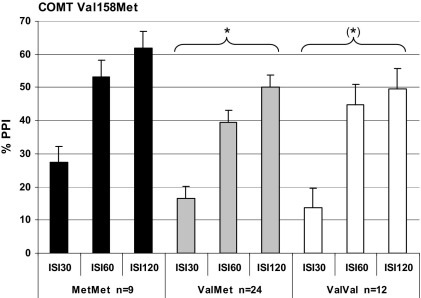
The effects of catechol-*O*-methyltransferase (COMT) Val158Met genotype on percent prepulse inhibition (PPI) of the acoustic startle response at prepulse (onset)-to-pulse (onset) intervals of 30, 60, and 120 msec in 45 healthy human male volunteers (means and SEM, adjusted for smoking; Tukey honest significant difference post hoc test versus the MetMet-group. (*)*p* = .08, *p < .05).

The Met homozygotes displayed the highest PPI levels but a 3 × 3 (SOA condition × genotype) repeated-measures ANCOVA with sex and smoking as covariates revealed only significant main effects for the factors SOA condition [*F*(2,87) = 5.5; *p* < .01; η^2^ = .11] and sex [*F*(1,88) = 13.7; *p* < .001; η^2^ = .13; see Figure 1B]. Given that Roussos *et al.* (12) showed their significant effects of COMT genotype on PPI in male subjects only, we excluded females from the ANCOVA analysis in a further step. Although only 45 males remained, this resulted in a significant main effect of genotype [*F*(2,41) = 3.2; *p* < .05; η^2^ = .13; see Figure 2]. Tukey HSD post hoc tests revealed that Met homozygotes displayed significantly higher PPI levels compared with heterozygotes (*p* < .05; *d* = .83). Both homozygous groups did not significantly diverge with respect to PPI although the difference did show a moderate effect size (*d* = .55). The ValVal group and the ValMet group did not differ. The male COMT genotype groups still did not differ in startle reactivity and habituation measures. Moreover, if male Met homozygotes were compared with a merged group of male carriers of the Val Allel (ValMet + ValVal) in a 3 × 2 (SOA condition × genotype) repeated-measures ANCOVA with smoking as a covariate, the Met homozygotes still showed significantly higher PPI levels that Val allele carriers [*F*(1,42) = 6.3, *p* < .05, η^2^ = .13].

### NRG-1 Arg38Gln Polymorphism

The NRG-1 Arg38Gln genotype frequencies were distributed in accordance with HWE [χ^2^(1) = .04; *p* = .84]. The three genotype groups did not differ regarding demographic characteristics, pooled PPI scores, startle reactivity, and startle latency measures (see Table 3), but there was a trend for a different distribution of sex between the genotype groups. Moreover, there was a strong trend for a genotype effect regarding early habituation (see Table 3). The total habituation and the slope of habituation revealed a similar but also nonsignificant pattern of effect as observed for early habituation.

Although the A homozygotes showed somewhat higher PPI levels, a 3 × 3 (SOA condition × genotype) repeated-measures ANCOVA with sex and smoking as covariates revealed significant main effects only for the factors SOA condition [*F*(2,93) = 9.0; *p* < .001; η^2^ = .16], and sex [*F*(1,94) = 18.1; *p* < .001; η^2^ = .16; see Figure 1C].

### Correlation Analysis and Genotype Interactions

Age and years of education did not correlate with any of the psychophysiologic parameters. A 3 × 3 × 3 (SOA condition × SNP × genotype) repeated-measures ANCOVA with the three SNPs under investigation and sex as covariate did not show any significant interactions between SNPs. Interaction analyses with two SNPs each did also not reveal any interactions. However, these analyses should be interpreted with caution because of the limited sample size.

## Discussion

This work aimed to replicate initial findings on the dependency of PPI on polymorphisms of the 5-HT_2A_ receptor, the COMT enzyme, and the NRG-1 signal protein in an independent sample of healthy human volunteers. First, we confirmed that the 5-HT_2A_R T102C/A-1438 G polymorphism exerts the same impact on sensorimotor gating in healthy humans as was previously shown in schizophrenia patients (14). Carriers of the C102/G-1438 alleles exhibited a significantly lower PPI than subjects homozygous for the T102/A-1438 alleles. Interestingly, the PPI variance explained by these 5-HT_2A_R SNPs was comparable between studies (schizophrenia patients: 11%; healthy control subjects: 10%), although we used different PPI paradigms and recruited the subjects in different European countries. Second, we were able to replicate the finding that male COMT Val158Met Met homozygotes display elevated PPI levels (12), but we were unable to detect this effect in our total mixed-sex sample. Roussos *et al.* (12) reported their COMT effects on PPI from a sample of male students, and we initially confirmed this effect for schizophrenia patients in a sample consisting of nearly 70% males (13). The fact that one study did not find an impact of that polymorphism on PPI in female subjects (27) further supports the notion that the COMT Val158Met genotype might affect PPI only in male subjects. Furthermore, the COMT Met158 allele has also been reported to have a greater impact on cognitive functions and PPI-related personality traits in males than females (28,29). The explained PPI variance by COMT genotype for the males in our study was 13%, which is situated between the effect in Roussos male sample (25%) and our mixed-sex schizophrenia sample (9%). Third, we could not replicate the finding of Hong *et al.* (11), who reported a moderate impact of the NRG-1 Arg38Gln polymorphism on PPI previously (7.9% variance explanation by NRG-1 genotype). In contrast to the lowered PPI levels in the homozygous A allele carriers in Hong's sample, we rather found slightly elevated PPI levels in this group. Thus, it is unlikely that our study was simply underpowered to generate the same effect. The mixed sample of Hong *et al.* (11) was indeed larger than ours but was heterogeneous, consisting of Caucasian and African Americans, schizophrenia patients, and healthy control subjects, whereas our sample includes exclusively Caucasian healthy control subjects. Finally, Hong *et al.* assessed PPI only at an SOA of 120 msec, and they used a slightly weaker prepulse intensity (80 vs. 85 dB). However, a selective analysis of our 120 msec SOA condition still revealed a different PPI pattern (AA > GG > AG), which could not be explained by the slightly different prepulse intensity. Thus, the discrepant results may be most likely caused by different sample compositions.

Importantly, our finding on the 5-HT_2A_R polymorphisms match nearly all criteria for a replication of a genotype–phenotype association proposed by the National Cancer Institute–National Human Genome Research Institute (NCI-NHGRI) Working Group on Replication in Association Studies (19): 1) we used a larger size than reported in the initial report, 2) we generated an independent data set, 3) we assessed the same phenotype, 4) we found similar effect sizes in the same SNPs, 5) we used the same statistical tests, 6) we had a rationale for reassessing these SNPs (susceptibility genes of schizophrenia), and 7) we provide at least the same level of detail for study design, analysis, and sample characteristics as reported in the initial study. Moreover, all analyzed genotype frequencies were distributed in accordance to the HWE so that genetic inhomogeneity of the investigated population is unlikely.

Our meta-analyses of the SchizophreniaGene online database revealed that among the four genetic variants investigated in our study, only the 5-HT_2A_R A-1438 G polymorphism is likely associated with the risk for schizophrenia (5-HT_2A_R A-1438 G: odds ratio in Caucasians [OR] = 1.17; COMT Val158Met: OR = 1.01; NRG-1 Arg38Gln: OR = .97; http://www.schizophreniaforum.org) (18). Lower PPI in carriers of the 5-HT_2A_R A-1438 G-G variant would therefore be consistent with the significant association of this allele with schizophrenia and the well-known PPI deficits in schizophrenia. The 5-HT_2A_R T102C and the A-1438 G polymorphism are silent mutations, but both may alter promoter activity and expression of 5-HT_2A_Rs (30,31). Thus, the C allele of the T102C variation or the G allele of the A-1438-G variation (or both) may cause lower 5-HT_2A_R densities in several brain areas that are involved in the processing of sensorimotor gating (14,32). Additionally, PPI deficits have also been reported in obsessive-compulsive disorder (33,34) and autism (35,36), in which both the 5-HT_2A_R A-1438 G polymorphism have been implicated (37,38). Then again, although local administration of dopamine agonists, antagonists, or depleters in the prefrontal cortex (PFC) disrupts PPI (39–43), there is some indirect evidence that the COMT antagonist tolcapone, which slightly increases dopamine in the PFC, improves PPI in COMT Val158-homozygotes (44). Thus, the increased PPI in male Met158 homozygotes could possibly be explained by a slight increase of dopamine in the PFC. However, more research is required to clarify the neurobiological basis of the COMT-genotype effect on PPI.

In conclusion, our findings support the view that sensorimotor gating is strongly modulated by 5-HT_2A_R A-1438 G/T102C genotype independent of sex, whereas the COMT Val158Met genotype only influences PPI in male subjects. In concert with previous human and animal findings showing that PPI is affected by multiple mutations, it is suggested that PPI (like schizophrenia) is modulated by polygenetic factors. Future studies with larger samples are needed to explore the multiple single and epistatic effects of different gene mutations on PPI, which may provide also windows into the polygenetic causation of schizophrenia.

## Figures and Tables

**Table 1:** Demographic Data and Psychophysiological Parameters of Healthy Human Volunteers Grouped According to Their 5-HT_2A_ A-1438G and T102C Receptor Genotype

5-HT2A A-1,438 G Genotype (rs6311)	AA	AG	GG					
5-HT2A T102C Genotype (rs6313)	TT	TC	CC	Total	*F*/χ^2^	*df*/*df*_err_	*p*	η^2^
*N*	19 (19.6%)	42 (43.3%)	36 (37.1%)	97 (100%)				
Age	26.8 (1.7)	25.8 (.9)	26.1 (.9)	26.1 (.6)	.20	2/96	.82	.00
Years of Education	16.8 (.9)	16.7 (.4)	17.8 (.6)	17.1 (.3)	1.35	2/96	.26	.03
Men, %	36.8	57.1	44.4	48.5	2.53	2	.28	—
Smokers, %	36.8	21.4	27.8	26.8	1.61	2	.45	—
First Block, Amplitude of Pulse-Alone Trials^a^ (Arbitrary Units)	742 (95.3)	712 (64.1)	701 (68.5)	714 (41.0)	.06	2/92	.94	.00
Mean Amplitude of Pulse-Alone Trials^a^ (Arbitrary Units)	561 (84.3)	578 (56.7)	590 (60.6)	580 (36.4)	.04	2/92	.96	.00
Mean Percent Prepulse Inhibition^a^ (Mean Across Three SOA Conditions)	41.3 (3.3)	28.3 (2.2)	31.2 (2.4)	31.9 (1.6)	5.28	2/92	.007^b^	.10
Percent Early Habituation of Pulse-Alone Trials^a^ (Between First and Second Block)	19.2 (6.3)	23.6 (4.2)	16.9 (4.5)	20.2 (2.7)	.61	2/92	.55	.01
Percent Total Habituation of Pulse-Alone Trials^a^ (Between First and Last Block)	34.2 (7.6)	28.9 (5.1)	26.0 (5.5)	28.9 (3.3)	.38	2/92	.98	.01
Habituation of Pulse-Alone Trials Across Four Blocks^a^ (Linear Gradient Coefficient *b*)	−90.8 (19.4)	−65.0 (13.0)	−60.5 (14.0)	−68.4 (8.4)	.86	2/92	.43	.02

5-HT_2A_, serotonin-2A receptor; SOA, stimulus-onset asynchronies. Means and SEM in parentheses, adjusted for sex and smoking; sex and smoking in frequency data. 5-HT_2A_ A-1438G and T102C receptor polymorphisms were in complete linkage disequilibrium.

a Analysis of covariance, means adjusted by covariates sex and smoking.

b Significant *p* values.

**Table 2:** Demographic Data and Psychophysiological Parameters of Healthy Human Volunteers Grouped According to Catechol-*O*-methyltransferase (COMT) Val158Met Genotype

COMT Val158Met Genotype (rs4680)	MetMet	ValMet	ValVal	Total	*F*/χ^2^	*df*/*df*_err_	*p*	η^2^
*n*	22 (23.7%)	53 (57.0%)	18 (19.4%)	93 (100%)				
Age	25.9 (1.3)	26.4 (.9)	25.7 (.9)	26.2 (.6)	.13	2/92	.88	.00
Years of Education	16.3 (.7)	17.1 (.4)	17.5 (.8)	17.0 (.3)	.79	2/92	.46	.02
Men, %	54.5	45.3	50.0	48.4	.56	2	.76	—
Smoker, %	27.3	28.3	16.7	25.8	.98	2	.61	—
First Block, Amplitude of Pulse-Alone Trials^a^ (Arbitrary Units)	775 (88.1)	685 (56.8)	749 (68.5)	718 (42.1)	.42	2/88	.66	.01
Mean Amplitude of Pulse-Alone Trials^a^ (Arbitrary Units)	644 (76.6)	561 (49.4)	559 (60.6)	580 (36.7)	.45	2/88	.64	.01
Mean Percent Prepulse Inhibition^a^ (Mean Across Three SOA Conditions)	35.8 (3.2)	30.2 (2.1)	34.3 (3.5)	32.3 (1.7)	1.28	2/88	.28	.03
Percent Early Habituation of Pulse-Alone Trials^a^ (Between First and Second Block)	14.7 (5.8)	21.5 (3.8)	26.0 (4.5)	20.6 (2.8)	.89	2/88	.41	.02
Percent Total Habituation of Pulse-Alone Trials^a^ (Between First and Last Block)	28.7 (7.0)	26.0 (4.5)	35.8 (7.7)	28.6 (3.3)	.60	2/88	.55	.01
Habituation of Pulse-Alone Trials Across Four Blocks^a^ (Linear Gradient Coefficient *b*)	−74.2 (17.8)	−57.1 (11.5)	−97.9 (19.7)	−69.1 (8.6)	1.65	2/88	.20	.04

SOA, stimulus-onset asynchronies.

a Analysis of covariance, means adjusted by covariates sex and smoking.

**Table 3:** Demographic Data and Psychophysiological Parameters of Healthy Human Volunteers Grouped According to Their Neuregulin-1 (NRG-1) Arg38Gln Genotype

NRG-1 Arg38Gln Genotype (rs3924999)	AA	AG	GG	Total	*F*/χ^2^	*df*/*df*_err_	*p*	η^2^
*n*	13 (13.1%)	47 (47.5%)	39 (39.4%)	99 (100%)				
Age	26.9 (1.9)	25.9 (.9)	26.1 (.8)	26.1 (.6)	.15	2/98	.86	.00
Years of Education	17.2 (.6)	16.8 (.4)	17.4 (.6)	17.1 (.3)	.41	2/98	.66	.01
Men, %	30.8	42.6	61.5	48.5	4.96	2	.08	—
Smokers, %	7.7	29.8	28.2	26.3	2.69	2	.26	—
First Block, Amplitude of Pulse-Alone Trials^a^ (Arbitrary Units)	848 (114.7)	684 (59.6)	702 (66.0)	713 (40.6)	.82	2/94	.45	.02
Mean Amplitude of Pulse-Alone Trials^a^ (Arbitrary Units)	650 (101.7)	573 (52.8)	554 (58.5)	576 (36.0)	.33	2/94	.72	.01
Mean Percent Prepulse Inhibition^a^ (Mean Across Three SOA Conditions)	35.6 (4.3)	30.7 (2.2)	32.2 (2.4)	32.0 (1.6)	.52	2/94	.60	.01
Percent Early Habituation of Pulse-Alone Trials^a^ (Between First and Second Block)	25.6 (7.5)	14.0 (3.9)	27.9 (4.3)	21.0 (2.7)	3.08	2/94	.05	.06
Percent Total Habituation of Pulse-Alone Trials^a^ (Between First and Last Block)	46.3 (9.0)	23.6 (4.7)	31.0 (5.2)	29.5 (3.2)	2.59	2/94	.08	.05
Habituation of Pulse-Alone Trials Across 4 Blocks^a^ (Linear Gradient Coefficient *b*)	−113.5 (23.1)	−57.6 (12.0)	−67.8 (13.3)	−69.0 (8.3)	2.31	2/94	.11	.05

Means and standard error of means in parentheses, adjusted for sex and smoking; sex and smoking in frequency data. SOA, stimulus-onset asynchronies.

a Analysis of covariance, means adjusted by covariates sex and smoking.

## References

[1] Swerdlow N.R., Geyer M.A. (1998). Using an animal model of deficient sensorimotor gating to study the pathophysiology and new treatments of schizophrenia. Schizophr Bull.

[2] Graham F.K. (1975). The more or less startling effects of weak prestimulation. Psychophysiology.

[3] Gottesman G.T.D. (2003). The endophenotype concept in psychiatry: Etymology and strategic intentions. Am J Psychiatry.

[4] Braff D.L., Geyer M.A., Swerdlow N.R. (2001). Human studies of prepulse inhibition of startle: Normal subjects, patient groups, and pharmacological studies. Psychopharmacology.

[5] Cadenhead K.S., Swerdlow N.R., Shafer K.M., Diaz M., Braff D.L. (2000). Modulation of the startle response and startle laterality in relatives of schizophrenic patients and in subjects with schizotypal personality disorder: Evidence of inhibitory deficits. Am J Psychiatry.

[6] Kumari V., Das M., Zachariah E., Ettinger U., Sharma T. (2005). Reduced prepulse inhibition in unaffected siblings of schizophrenia patients. Psychophysiology.

[7] Quednow B.B., Frommann I., Berning J., Kühn K.-U., Maier W., Wagner M. (2008). Impaired sensorimotor gating of the acoustic startle response in the prodrome of schizophrenia. Biol Psychiatry.

[8] Dulawa S.C., Geyer M.A. (2000). Effects of strain and serotonergic agents on prepulse inhibition and habituation in mice. Neuropharmacology.

[9] Willott J.F., Tanner L., O'Steen J., Johnson K.R., Bogue M.A., Gagnon L. (2003). Acoustic startle and prepulse inhibition in 40 inbred strains of mice. Behav Neurosci.

[10] Anokhin A.P., Heath A.C., Myers E., Ralano A., Wood S. (2003). Genetic influences on prepulse inhibition of startle reflex in humans. Neurosci Lett.

[11] Hong L.E., Wonodi I., Stine O.C., Mitchell B.D., Thaker G.K. (2008). Evidence of missense mutations on the neuregulin 1 gene affecting function of prepulse inhibition. Biol Psychiatry.

[12] Roussos P., Giakoumaki S.G., Rogdaki M., Pavlakis S., Frangou S., Bitsios P. (2008). Prepulse inhibition of the startle reflex depends on the catechol O-methyltransferase Val158Met gene polymorphism. Psychol Med.

[13] Quednow B.B., Wagner M., Mossner R., Maier W., Kuhn K.U. (2008). Sensorimotor gating of schizophrenia patients depends on catechol O-methyltransferase Val158Met polymorphism [published online ahead of print July 17]. Schizophr Bull.

[14] Quednow B.B., Kuhn K.U., Mossner R., Schwab S.G., Schuhmacher A., Maier W. (2008). Sensorimotor gating of schizophrenia patients is influenced by 5-HT2A receptor polymorphisms. Biol Psychiatry.

[15] Roussos P., Giakoumaki S.G., Bitsios P. (2008). The dopamine D(3) receptor Ser9Gly polymorphism modulates prepulse inhibition of the acoustic startle reflex. Biol Psychiatry.

[16] Abdolmaleky H.M., Faraone S.V., Glatt S.J., Tsuang M.T. (2004). Meta-analysis of association between the T102C polymorphism of the 5HT2A receptor gene and schizophrenia. Schizophr Res.

[17] Harrison P.J., Weinberger D.R. (2005). Schizophrenia genes, gene expression, and neuropathology: On the matter of their convergence. Mol Psychiatry.

[18] Allen N.C., Bagade S., McQueen M.B., Ioannidis J.P., Kavvoura F.K., Khoury M.J. (2008). Systematic meta-analyses and field synopsis of genetic association studies in schizophrenia: The SzGene database. Nat Genet.

[19] Chanock S.J., Manolio T., Boehnke M., Boerwinkle E., Hunter D.J., Thomas G. (2007). Replicating genotype–phenotype associations. Nature.

[20] Freeman B., Smith N., Curtis C., Huckett L., Mill J., Craig I.W. (2003). DNA from buccal swabs recruited by mail: Evaluation of storage effects on long-term stability and suitability for multiplex polymerase chain reaction genotyping. Behav Genet.

[21] Myakishev M.V., Khripin Y., Hu S., Hamer D.H. (2001). High-throughput SNP genotyping by allele-specific PCR with universal energy-transfer-labeled primers. Genome Res.

[22] Kumari V., Antonova E., Zachariah E., Galea A., Aasen I., Ettinger U. (2005). Structural brain correlates of prepulse inhibition of the acoustic startle response in healthy humans. Neuroimage.

[23] Kumari V., Gray J.A. (1999). Smoking withdrawal, nicotine dependence and prepulse inhibition of the acoustic startle reflex. Psychopharmacology.

[24] Quednow B.B., Wagner M., Westheide J., Beckmann K., Bliesener N., Maier W. (2006). Sensorimotor gating and habituation of the startle response in schizophrenic patients randomly treated with amisulpride or olanzapine. Biol Psychiatry.

[25] Swerdlow N.R., Hartman P.L., Auerbach P.P. (1997). Changes in sensorimotor inhibition across the menstrual cycle: Implications for neuropsychiatric disorders. Biol Psychiatry.

[26] Blumenthal T.D., Dawson M.E., Schell A.M., Böhmelt A.H. (1999). Short lead interval startle modification. Startle Modification Implications for Neuroscience, Cognitive Sciences, and Clinical Science.

[27] Montag C., Hartmann P., Merz M., Burk C., Reuter M. (2008). D(2) receptor density and prepulse inhibition in humans: Negative findings from a molecular genetic approach. Behav Brain Res.

[28] Harrison P.J., Tunbridge E.M. (2008). Catechol-O-methyltransferase (COMT): A gene contributing to sex differences in brain function, and to sexual dimorphism in the predisposition to psychiatric disorders. Neuropsychopharmacology.

[29] Talledo J.A., Sutherland Owens A.N., Schortinghuis T., Swerdlow N.R. (2009). Amphetamine effects on startle gating in normal women and female rats. Psychopharmacology.

[30] Parsons M.J., D'Souza U.M., Arranz M.J., Kerwin R.W., Makoff A.J. (2004). The -1438 amperes/G polymorphism in the 5-hydroxytryptamine type 2A receptor gene affects promoter activity. Biol Psychiatry.

[31] Serretti A., Drago A., De Ronchi D. (2007). HTR2A gene variants and psychiatric disorders: A review of current literature and selection of SNPs for future studies. Curr Med Chem.

[32] Vollenweider F.X., Csomor P.A., Knappe B., Geyer M.A., Quednow B.B. (2007). The effects of the preferential 5-HT2A agonist psilocybine on prepulse inhibition of startle in healthy human volunteers depend on interstimulus interval. Neuropsychopharmacology.

[33] Hoenig K., Hochrein A., Quednow B.B., Maier W., Wagner M. (2005). Impaired prepulse inhibition of acoustic startle in obsessive-compulsive disorder. Biol Psychiatry.

[34] Swerdlow N.R., Benbow C.H., Zisook S., Geyer M.A., Braff D.L. (1993). A preliminary assessment of sensorimotor gating in patients with obsessive compulsive disorder. Biol Psychiatry.

[35] McAlonan G.M., Daly E., Kumari V., Critchley H.D., van Amelsvoort T., Suckling J. (2002). Brain anatomy and sensorimotor gating in Asperger's syndrome. Brain.

[36] Perry W., Minassian A., Lopez B., Maron L., Lincoln A. (2007). Sensorimotor gating deficits in adults with autism. Biol Psychiatry.

[37] Cho I.H., Yoo H.J., Park M., Lee Y.S., Kim S.A. (2007). Family-based association study of 5-HTTLPR and the 5-HT2A receptor gene polymorphisms with autism spectrum disorder in Korean trios. Brain Res.

[38] Walitza S., Wewetzer C., Warnke A., Gerlach M., Geller F., Gerber G. (2002). 5-HT2A promoter polymorphism -1438 G/amperes in children and adolescents with obsessive-compulsive disorders. Mol Psychiatry.

[39] Broersen L.M., Feldon J., Weiner I. (1999). Dissociative effects of Apomorphine infusions into the medial prefrontal cortex of rats on latent inhibition, prepulse inhibition and amphetamine-induced locomotion. Neuroscience.

[40] Bubser M., Koch M. (1994). Prepulse inhibition of the acoustic startle response of rats is reduced by 6-hydroxydopamine lesions of the medial prefrontal cortex. Psychopharmacology.

[41] Ellenbroek B.A., Budde S., Cools A.R. (1996). Prepulse inhibition and latent inhibition: The role of dopamine in the medial prefrontal cortex. Neuroscience.

[42] Lacroix L., Broersen L.M., Feldon J., Weiner I. (2000). Effects of local infusions of dopaminergic drugs into the medial prefrontal cortex of rats on latent inhibition, prepulse inhibition and amphetamine induced activity. Behav Brain Res.

[43] Zavitsanou K., Cranney J., Richardson R. (1999). Dopamine antagonists in the orbital prefrontal cortex reduce prepulse inhibition of the acoustic startle reflex in the rat. Pharmacol Biochem Behav.

[44] Giakoumaki S.G., Roussos P., Bitsios P. (2008). Improvement of prepulse inhibition and executive function by the COMT inhibitor tolcapone depends on COMT Val158Met polymorphism. Neuropsychopharmacology.

